# Evaluating deep learning EEG-based mental stress classification in adolescents with autism for breathing entrainment BCI

**DOI:** 10.1186/s40708-021-00133-5

**Published:** 2021-07-13

**Authors:** Avirath Sundaresan, Brian Penchina, Sean Cheong, Victoria Grace, Antoni Valero-Cabré, Adrien Martel

**Affiliations:** 1 The Nueva School, San Mateo, CA 94033 USA; 2 Muvik Labs, LLC, Locust Valley, NY 11560 USA; 3 Center for Computer Research in Music and Acoustics, Stanford University, Stanford, CA 94305 USA; 4 Department of Anatomy and Neurobiology, Laboratory of Cerebral Dynamics, Boston University School of Medicine, Boston, MA 02118 USA; 5 Cognitive Neuroscience and Information Technology Research Program, Open University of Catalonia (UOC), Barcelona, Spain; 6 Causal Brain Dynamics, Plasticity and Rehabilitation Team, Frontlab, Brain and Spine Institute, ICM, CNRS UMR, Paris, 7225 France

**Keywords:** Mental stress, Autism, EEG, Deep learning, Breathing entrainment

## Abstract

Mental stress is a major individual and societal burden and one of the main contributing factors that lead to pathologies such as depression, anxiety disorders, heart attacks, and strokes. Given that anxiety disorders are one of the most common comorbidities in youth with autism spectrum disorder (ASD), this population is particularly vulnerable to mental stress, severely limiting overall quality of life. To prevent this, early stress quantification with machine learning (ML) and effective anxiety mitigation with non-pharmacological interventions are essential. This study aims to investigate the feasibility of exploiting electroencephalography (EEG) signals for stress assessment by comparing several ML classifiers, namely support vector machine (SVM) and deep learning methods. We trained a total of eleven subject-dependent models-four with conventional brain-computer interface (BCI) methods and seven with deep learning approaches-on the EEG of neurotypical (*n*=5) and ASD (*n*=8) participants performing alternating blocks of mental arithmetic stress induction, guided and unguided breathing. Our results show that a multiclass two-layer LSTM RNN deep learning classifier is capable of identifying mental stress from ongoing EEG with an overall accuracy of 93.27%. Our study is the first to successfully apply an LSTM RNN classifier to identify stress states from EEG in both ASD and neurotypical adolescents, and offers promise for an EEG-based BCI for the real-time assessment and mitigation of mental stress through a closed-loop adaptation of respiration entrainment.

## Introduction

Individuals with autism spectrum disorder (ASD) often demonstrate deficits in social communication skills and restricted or stereotyped behaviors and interests [1]. This causes those with ASD to experience states of cognitive and emotional overload, leading to increased stress and ultimately anxiety symptoms [2]. Although there is significant overlap between stress and anxiety, stress is best understood as the physiological and psychological response towards stressors; anxiety is the persistence of stress even in the absence of these stressors. The comorbidity of anxiety disorders and ASD in children and adolescents has been studied extensively with 40% to 85% of individuals with ASD aged 6 to 18 having at least one form of anxiety [3–5]. Unfortunately, individuals with ASD are uniquely vulnerable to the deleterious effects of stress because of their hyper- or hyporeactivity to sensory inputs, as well as difficulties with accurate stress detection and coping with stressful situations [6]. Given the frequency in which anxiety co-occurs in ASD, in conjunction with the hurdles in education, long-term functional impairments, reduction in quality of life, and increased caregiver burden [7–13], a more comprehensive understanding of comorbidities in ASD as well as personalized intervention methods to relieve clinical symptoms of the disease and improve emotional and physical well-being for individuals with ASD is needed. Incidentally, anxiety and the design of appropriate intervention methods have been identified by the autism community and clinicians as a key priority with researchers emphasizing the need for more precise measures of anxiety [14]. Moreover, the lack of objective and continuous measurements of stress is particularly detrimental for a population already affected by an inability to express inner experiences and calls for novel methods to identify individualized stress markers in real-time [15]. Among the triggers identified, such as challenging sensory experiences or social demands, anxiety related to academic expectations is thought to have the greatest impact on school performance for ASD children and adolescents [16, 17].

Concurrently, a growing number of studies have demonstrated the efficacy of stand-alone meditation, relaxation and breathing practices for improving well-being, mental health and managing stress [18–20]. Although the underlying mechanisms are not yet fully understood, breathing practices such as ‘anatomically optimized respiration’, i.e. controlled, slow diaphragmatic breathing through the nose in the range of 6-10 breaths per minute (brpm) or resonance frequency breathing, 4.5 to 6.5 brpm for adults and 6.5 to 9.5 brpm for children, have been found to procure significant physiological benefits [21, 22], reduce physiological and psychological stress [23] and even improve sustained attention performance [24]. Prior studies have shown that breath-control can address physiological correlates of anxiety, including heart rate variability [22, 25], a well-validated quantitative stress indicator [26]. Notably, breath-control has been found to significantly decrease test anxiety in students in an educational setting [27]. Moreover, a recent review by Zaccaro et al. [28] found that controlled slow breathing (<10 brpm) had a significant impact on autonomic nervous system activity, especially in the theta (3–7 Hz), alpha (8–14 Hz) and beta (15–30Hz) bands of the electroencephalogram (EEG), linked to improved cognitive performance during attentional and executive functions [29]. Although these findings, taken together, speak to the promise of using controlled slow breathing as a simple, low-cost and non-pharmacologic intervention [23] to mitigate anxiety, optimized efficacy hinges on assessing an individual’s current level of stress and ideal respiration parameters in real-time.

Although cognitive or affective states such as stress are not directly observable externally nor reliably measurable through behavioral measures or subjective reports, developments in EEG-based brain-computer interfaces (BCIs) have increasingly permitted the continuous and real-time monitoring of mental states. Neuroadaptive technologies and passive brain-computer interfaces (pBCIs) aim at intelligent forms of adaptation in response to cognitive state assessments [30, 31]. The field of EEG-based BCIs has blossomed in recent years, largely on account of EEG’s high temporal resolution, non-invasiveness, relatively low cost, and novel advances in the effectiveness and usability of acquisition systems [32, 33]. While BCIs have historically been employed in the context of assistive technologies for severely impaired individuals [34, 35], pBCIs have mainly been aimed at developing adaptive automation for real-world applications [36–38]. The central challenge of EEG-based pBCIs is to account for the high inter- and intra-individual variability of neurophysiological signals exhibited under particular cognitive states [39]. However, by averaging over a large enough number of samples it is possible to distill sufficiently specific brain activity patterns and train a machine learning classifier to learn to discern these patterns in real-time [40, 41]. This approach has already been successfully applied to monitor several cognitive states such as workload [42–44], vigilance [45–47], and fatigue [48, 49].

Neurofeedback involves monitoring a user’s mental state with EEG and providing feedback through a variety of modalities (visual, audio, tactile, etc.) to modulate particular biomarkers [50]. In conjunction with breath-control, neurofeedback training has been shown to be a promising mitigatory tool for anxiety. For example, White et al. [51] demonstrated that breathing-based visual neurofeedback reduces symptoms in patients with anxiety and depression, while acoustically mediated deep breathing neurofeedback was shown by Crivelli et al. [52] to diminish long-term stress and anxiety levels in young adults. The first step towards an EEG-based BCI able to monitor anxiety levels, identify an individual’s optimal breathing patterns, and adapt breathing entrainment parameters in real-time, is to determine whether mental stress can be classified on the basis of ongoing EEG data.

Classification algorithms are key elements of any EEG-based BCI’s ability to recognize users’ EEG patterns and associated cognitive states. Among the large diversity of existing architectures and types of classifiers (for reviews see [53] and [54]), deep learning methods have recently emerged as methods of analysis that can consider neurophysiological data in its entirety, including the time domain [54]. Convolutional neural networks are the most widely used deep learning algorithms in EEG analysis [55], and have been shown to be effective in emotion detection [56, 57] and anxiety classification [58] in particular. Further, deep learning with convolutional neural networks (CNNs) have recently been shown to outperform the widely used filter bank common spatial pattern (FBCSP) algorithm [59] by extracting increasingly more complex features of the data [60]. Accordingly, we aimed at comparing several classifiers previously used in EEG-based BCIs for the classification of different states of anxiety in ASD and neurotypical adolescents. We employed classical machine learning methods, specifically support vector machines (SVMs) combined with FBCSP, which have been successfully applied to detect a wide range of covert cognitive and emotional states [53], including mental stress detection [61–64]. Although classical classifiers present several drawbacks compared to deep learning (e.g. elaborate feature extraction and extensive prior knowledge about the dataset [55, 65]), SVMs remain a useful benchmark against which deep learning methods can be evaluated.

For deep learning methods we selected EEGNet, a recently developed compact CNN for EEG-based BCIs [66], as well as the Deep ConvNet and the Shallow ConvNet developed by Schirrmeister et al. [60]. Moreover, we also applied long short-term memory recurrent neural networks (LSTM RNNs), which are a type of neural net with the ability to “remember” long-term dependencies far better than traditional RNNs, without the loss of short-term memory [67], and enable robust analysis of temporal trends in EEG data [68]. LSTM RNNs have also shown high accuracy in emotion detection [69], with LSTM RNN architectures performing better than CNN architectures on DEAP, a major EEG emotion analysis dataset [55]. Hybrid deep neural networks combining both LSTM RNN and CNN architectures have also shown promising results on DEAP [70, 71]. Building upon these recent advancements, we implemented an LSTM RNN and a hybrid long short-term memory fully convolutional network (LSTM-FCN) [72] to classify states of mental stress from EEG.

The primary purpose of the present study is to assess the feasibility of real-time anxiety detection based on EEG signals and the identification of a robust classifier for prospective use in a pBCI able to identify the optimal breathing patterns and alleviate anxiety in students with and without ASD. To our knowledge, this is the first study to examine the efficacy of deep learning-based EEG anxiety classifiers in comparison to classical methods. In addition, ours is the first attempt of EEG-based anxiety classification for both adolescents with autism and neurotypical adolescents.

## Methods

### Participants and data acquisition

Eight students (1 female M: 15.13 SD: 1.45) diagnosed with autism, designated as participants L1-L8, from Learning Farm Educational Resources based in Menlo Park, (California), and five students (1 female M: 16.6 SD: 0.55) with no known mental or neurological disorders, designated as participants T1-T5, from The Nueva School in San Mateo, (California), voluntarily enrolled in the study. Participants and their parents or legal guardians were informed extensively about the experiment and all gave written consent. The study was approved by an Institutional Review Board composed of an educator from Learning Farm Educational Resources, an administrator from The Nueva School, and a licensed mental health professional at The Nueva School.

Participants were seated in an isolated and dimly lit room at a viewing distance of approximately 70 cm of a 16” LCD monitor with a refresh rate of 60Hz. 16-channel EEG data were acquired at 125Hz using an OpenBCI system (Ag/AgCl coated electrodes + Cyton Board; https://openbci.com/) placed according to the international 10–20 system (channels: ‘Fp1’, ‘Fp2’, ‘C3’, ‘C4’, ‘P7’, ‘P8’, ‘O1’, ‘O2’, ‘F7’, ‘F8’, ‘F3’, ‘F4’, ‘T7’, ‘T8’, ‘P3’, ‘P4’). The 16-electrode OpenBCI apparatus was selected as it maximized portability, affordability, signal quality, and ease of use while minimizing the amount of electrodes, which is ideal for practical use of the mental stress detection system.

**Fig. 1:**
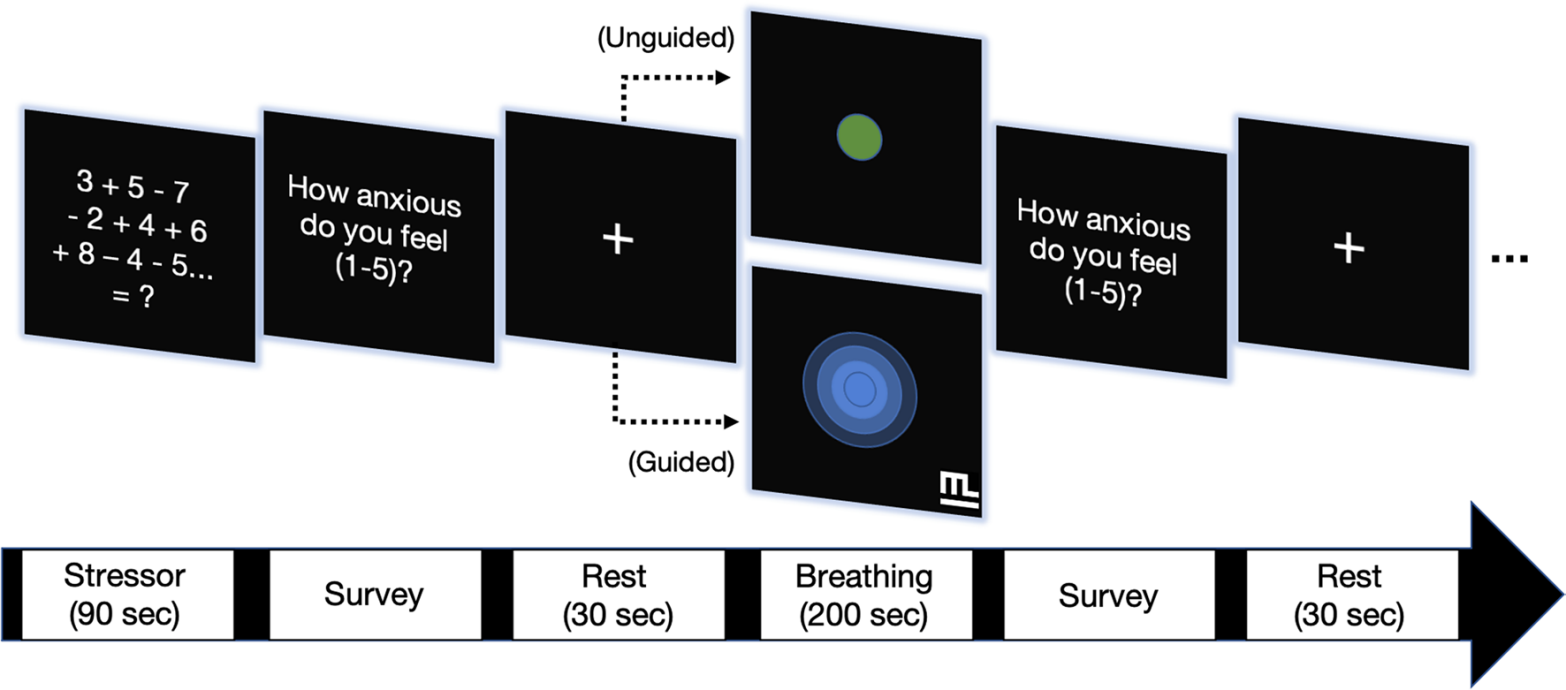
Experimental design of the procedure. Participants performed four blocks, each consisting of a mental arithmetic task followed by an anxiety self-report, a period of rest, either *guided breathing* entrainment or *unguided breathing*, a second anxiety self-report and lastly another rest period

Participants were fitted with passive noise-canceling headphones to isolate them from ambient noise and to interact with the stress and breath modulating interface. The audio-visual stimuli was designed in close collaboration with Muvik Labs (https://muviklabs.io). The stimuli featured sequential trials of stressor, guided breathing, and unguided breathing sections (Fig. 1). The stimuli were procedurally generated by Muvik Labs’ Augmented Sound $^{\mathrm{TM}}$ engine to ensure timing precision and effectiveness through evidence-backed breathing interventions driven by principles of psychoacoustics and behavioral psychology [73].

Prior to the main procedure, participants were asked to complete the trait anxiety component of Spielberger’s State-Trait Anxiety Inventory for Children (STAI-C) [74], a well-validated state and trait anxiety screen used for typically developing youth that can also be accurately used to assess trait anxiety in children and adolescents with autism [75].

### Stress induction and alleviation

Following an initial EEG baseline recording for 120 sec (‘Baseline’), participants performed a 25 min session featuring stress induction and breath modulation tasks consisting of four main blocks. Each block began with a stressor featuring an augmented arithmetic number task, intensified by bright contrasting colors displaying numbers appearing sequentially, coupled with audible sonified timers mapped to rising pitches similar to Shepard tones (powered by Muvik Labs Augmented Sound $^{\mathrm{TM}}$) [73], with a 90 second time constraint.

Timed mental arithmetic has been extensively used to induce stress [76, 77]; for our specific mental stress induction paradigm, we chose a widely used mental arithmetic task (for an overview see [63]) and simplified it to minimize the possibility of overstimulating participants with ASD. The mental stress induction was followed by a period of breathing for 200 seconds. The first and third breathing periods had participants breathe at their own pace (unguided breathing) while the second and fourth breathing periods presented participants with a custom-generated breathing entrainment system, guiding breath airflow in and out of lungs at a relaxing pace of around 6 brpm [78, 79] with both visual (i.e. growing/shrinking circle outlining the air flow volume of target respiration speed) and auditory guides (musical patterns featuring nature sounds that mimic the sound of inhalation and exhalation; Muvik Labs Augmented Sound $^{\mathrm{TM}}$). Following each mental arithmetic task and breathing period, participants were prompted to rate their current stress levels on a 5-point Likert scale, with 1 indicating “very relaxed” and 5 indicating “very stressed”.

### EEG signal processing and training data selection

MNE [80], an open-source Python tool for EEG analysis, was employed to filter EEG data from all 16 channels. In preparation for classification analysis, EEG time-courses were high-pass filtered at 1 Hz to remove slow trends and subsequently low-pass filtered at 50Hz to remove line noise. The routine clinical bandwidth for EEG is from 0.5 to 50Hz [81]. However, significant sinusoidal drift was observed on the 0.5Hz-1Hz interval and therefore the interval was excluded in the selected bandpass filter range.

The data of two participants were rejected from all analyses due to unusually high impedances at the time of recording, which was confirmed offline by visual inspection: participant L1 from the ASD group, and participant T3 from the neurotypical group. Preprocessing of the EEG data was kept to a minimum to mimic online conditions found in a real-time BCI scenario.

For training sample preparation, a cropped training strategy was employed. The number of samples extracted for the different classifiers are shown in Table 1. Samples with a length of 1 or 5s were extracted per participant from the EEG recorded during the ‘Stressor’, ‘Guided Breathing’, ‘Unguided Breathing’, and ‘Baseline’ periods of the procedure and were assigned corresponding labels.

**Fig. 2:**
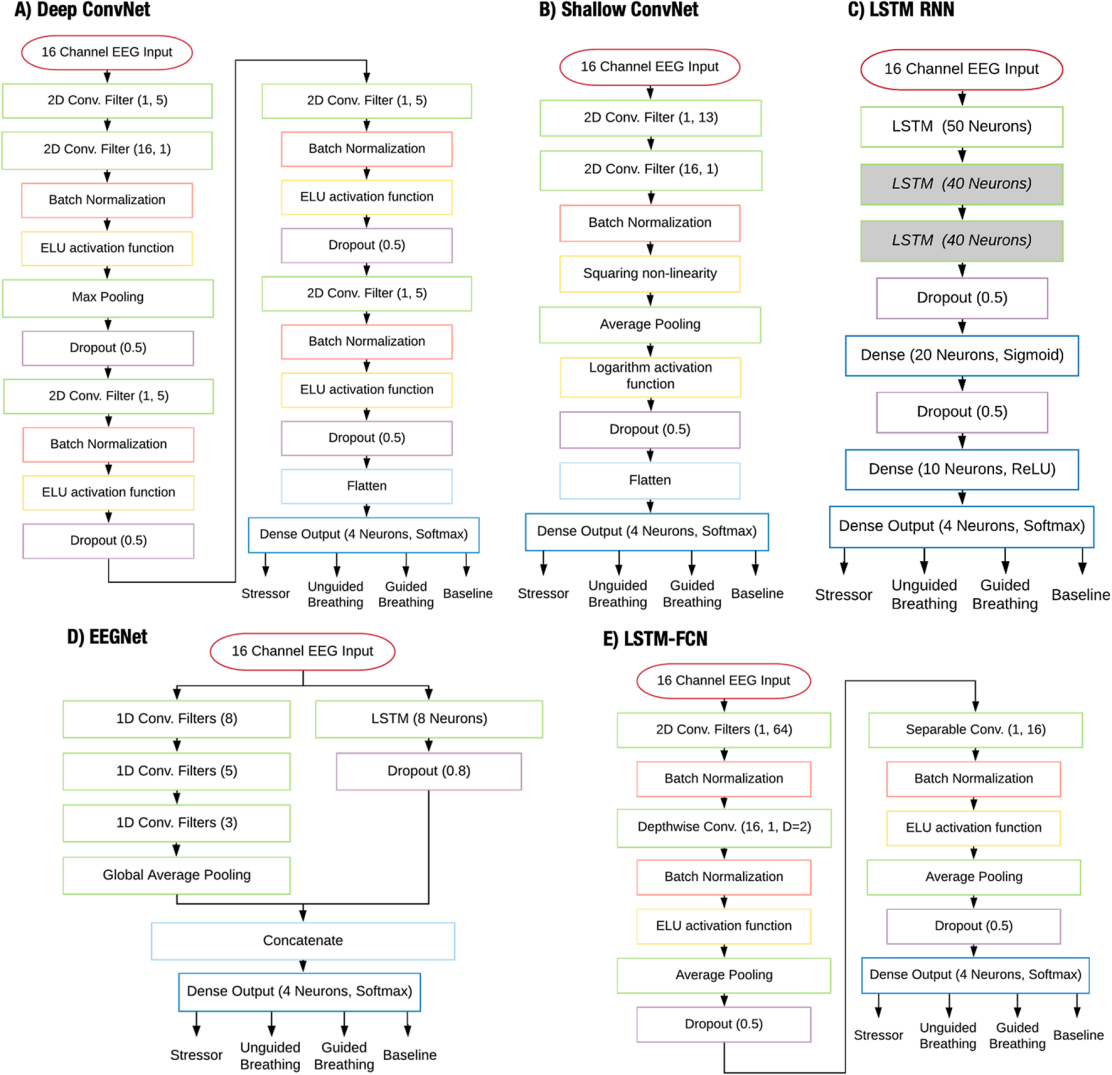
Diagram of the model architectures for the **A** Deep ConvNet, **B** Shallow ConvNet,** C** LSTM RNN,** D** EEGNet and** E** LSTM-FCN. Note: the first grayed layer of the LSTM RNN was only implemented for the two- and three-layer LSTM while the second grayed layer is only applicable to the three-layer LSTM

### Neural signal classification

We performed classification analysis on the selected EEG training samples using an SVM model with FBCSP, three CNN models, three LSTM RNN models, and a hybrid LSTM-FCN model. While all deep learning models were multiclass (‘Stressor’, ‘Baseline’, ‘Guided Breathing’ and ‘Unguided Breathing’), the SVM classifiers were binary (‘Guided Breathing’ vs ‘Stressor’, ‘Unguided Breathing’ vs ‘Stressor’, ‘Unguided Breathing’ vs ‘Guided Breathing’, & ‘Baseline’ vs ‘Stressor’), as is conventional for the classification of multiple classes with SVMs [82]. We opted to avoid using calculated features as inputs in favor of an end-to-end learning method with filtered EEG signal value inputs from all 16 channels. In addition, as different EEG channels represent neural signals from different areas of the brain, we elected not to combine channel data to preserve spatial information.

For the FBCSP-SVM, the EEG recording was divided in the time domain into samples of 1s and partitioned in the frequency domain using 9 filter bank band-pass filters from 4Hz to 40Hz prior to feature extraction, which was achieved with the common spatial pattern (CSP) algorithm, i.e. a linear map maximizing the variance difference between two classes [83]. The binary SVM classifiers used a radial basis function kernel with a gamma value of 1/360 and a regularization parameter (C) of 1.6. We implemented a number of FBCSP-SVM variants, such as multiclass SVM (baseline, stressor, guided and unguided breathing) with polynomial or sigmoid kernels and a 5 sec EEG sample length; these models were not included in the comparison due to lower classification performance. The FBCSP-SVMs were implemented with the sklearn library in Python and for validation samples were apportioned at a ratio of 80:20 for train and test dataset.

The Deep ConvNet CNN architecture [60] is composed of 4 convolution-max-pooling blocks. The first block, with 25 2D temporal convolutional filters of size (1, 5), 25 2D spatial convolutional filters of size (1, 64), and a max pooling layer, was especially designed to process the EEG input. The subsequent convolution-max-pooling blocks each have a 2D convolutional layer and a max pooling layer, with 50, 100 and 200 convolutional filters per block, respectively. Finally, a 4 neuron dense layer with softmax activation produces the output (see Fig. 2A). The Shallow ConvNet CNN architecture is a modification of the Deep ConvNet to mimic the transformations of FBCSP. The Shallow ConvNet retains the first convolution-max-pooling block of the Deep ConvNet, albeit with a larger kernel size of 13 for the temporal convolution layer. This block performs similar transformations as the bandpass filter and CSP spatial filtering algorithm of the FBCSP workflow. Following the convolution-max-pooling block, the architecture contains a squaring nonlinearity function, an average pooling layer, and a logarithm activation function [60]. A 4 neuron dense layer with softmax activation produces the output (see Fig. 2B).

We trained three original LSTM RNN models, with one, two and three LSTM layers, respectively. The three-layer LSTM RNN model consists of three LSTM layers, two dense hidden layers, and a dense output layer. The first LSTM layer, containing 50 neurons, receives the input. The second and third LSTM layers contain 40 neurons. The number of neurons in the LSTM layers was informed by the amount calculated and used by Tsiouris et al. [84] and Alhagry et al. [69], and adjusted to our EEG data to prevent underfitting and overfitting. Following the third LSTM layer, we include a dropout layer to reduce overfitting [85] with a dropout rate of 0.5. The first dense layer contains 20 neurons and uses a sigmoid activation function. Following the first dense layer, we include another dropout layer with a dropout rate of 0.5. The second dense layer consisted of 10 neurons and used a rectified linear unit (ReLU) as an activation function. The dense output layer of 4 neurons used softmax activation. The two-layer LSTM architecture is obtained by omitting one 40 neuron LSTM layer, and the one-layer LSTM architecture is obtained by omitting both 40 neuron LSTM layers (see Fig. 2C).

The EEGNet CNN architecture [66] used comprised 8 2D convolutional filters of size (1, 64), a Depthwise Convolution layer of size (16, 1) to learn multiple spatial filters for each temporal filter, a Separable Convolution layer of size (1, 16), and a 4 neuron dense layer with softmax activation (see Fig. 2D). In the LSTM-FCN [72] architecture, EEG time series input is simultaneously fed into an LSTM block, composed of an 8 neuron LSTM layer and a dropout layer with rate of 0.8, and an FCN block composed of 128 1D temporal convolutional layers of size 8, 256 1D temporal convolutional layers of size 5, and 128 1D temporal convolutional layers of size 3. The outputs of the LSTM and FCN blocks are then concatenated and passed into a 4 neuron dense output layer with softmax activation (see Fig. 2E).

All deep learning architectures were implemented with the Keras machine learning library in Python, and were trained over 1000 EEG epochs with a batch size of 200. While training, we implemented the Adam optimization algorithm [86] with a learning rate of 0.001 in place of the standard stochastic gradient descent (SGD) algorithm. During validation, EEG samples for deep learning were apportioned at a ratio of 70:30 to the train dataset and test dataset, respectively.

## Results

### Behavioral results

On average, participants self-reported higher levels of mental stress on the 5-point scale following the stress induction periods, with average stress scores of 1.54, 2.04, and 1.62 prior to 2nd, 3rd, and 4th stress induction periods, respectively, and average scores of 3.00, 2.88, and 3.12 following the same stress induction periods. A series of Wilcoxon signed-rank tests were employed to compare the self-reported stress scores before and after each stress induction period across all participants; the tests indicated that post-stressor stress scores were significantly higher than the pre-stressor scores, with Z test statistics of − 3.19, − 2.49, and − 2.62, and* p*-values of 0.00143, 0.0127, and 0.00879, for the 2nd, 3rd, and 4th stress induction periods, respectively. As the participants were prompted for their self-reported mental stress level following every stressor and breathing period, stress scores prior to the 1st stress induction period were not collected and hence the 1st stress induction period was not considered in the behavioral data analysis. Accompanied by the precedents in the literature, these results reinforce our confidence that the selected experimental paradigm can reliably induce mental stress in the participants.

### Model performance

The performance for the FBCSP-SVM classifiers of each participant are shown in Table 1. The average classification accuracy was highest for the binary classification of ‘Baseline’ vs ‘Stressor’ (87.88%) and lowest for ‘Unguided Breathing’ vs ‘Stressor’ (78.28%). That the baseline and unguided breathing conditions had the lowest level of demands imposed on participants, and yet classification against the stressor condition yielded both the highest and lowest accuracy, suggests that muscle activity did not bias the classifiers significantly.

**Table 1:** Classification accuracies of the FBCSP-SVM classifiers per participant and classification (‘Guided Breathing’ vs ‘Stressor’, ‘Baseline’ vs ‘Stressor’, ‘Unguided Breathing’ vs ‘Guided Breathing’, and ‘Guided Breathing’ vs ‘Stressor’)

Participant	Guided vs Stressor (%)	Baseline vs Stressor (%)	Unguided vs Guided (%)	Unguided vs Stressor (%)
L2	98.75	97.92	100.00	96.30
L3	80.00	95.83	75.00	80.56
L4	68.75	91.67	70.00	75.93
L5	81.25	91.67	87.50	77.78
L6	67.50	81.25	57.50	71.30
L7	87.50	81.25	97.50	90.74
L8	90.00	93.75	86.25	70.37
T1	90.00	93.75	76.25	71.30
T2	83.75	89.58	91.25	83.33
T4	72.50	79.17	73.75	69.44
T5	65.00	70.83	71.25	74.04
Average	80.45	87.88	80.57	78.28

The three CNN models, Deep ConvNet, Shallow ConvNet, and EEGNet, yielded average classification accuracies of 58.80, 62.84, and 61.18%, respectively (see Table 2). The LSTM-FCN yielded an average classification accuracy of 62.97% across all four classes. The two-layer LSTM RNN classifier yielded an average accuracy of 93.27% on the test data across all four classes, outperforming the 73.53% average accuracy of the one-layer LSTM RNN and the 76.06% average accuracy of the three-layer classifier (see Table 3).

**Fig. 3:**
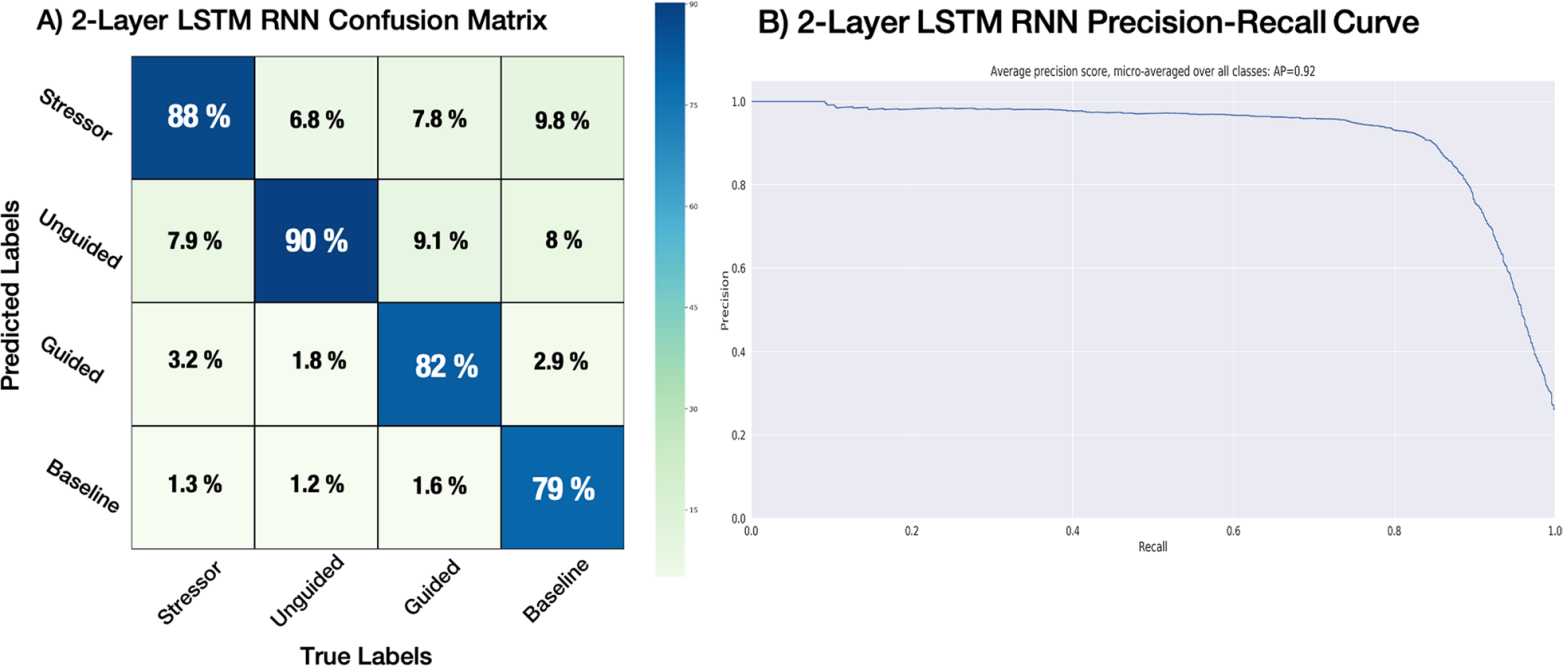
**A** 2-Layer LSTM RNN model confusion matrix.** B** 2-Layer LSTM RNN odel precision-recall curve

It is important to note that due to the longer length of the unguided and guided breathing periods compared to the stressor and the baseline periods, more samples were extracted from the unguided and guided breathing periods, creating an unbalanced dataset. Although this can lead to issues since an unbalanced dataset can artificially inflate the accuracy metric, the two-layer LSTM RNN model used here demonstrated high class-wise sensitivity and specificity during validation (see Fig. 3), leading us to the conclusion that the unbalanced dataset was not a cause for concern.

**Table 2:** Class-wise and overall accuracies for the Deep ConvNet, Shallow ConvNet, and EEGNet CNN classifiers

Average accuracy (%)	Deep ConvNet (%)	Shallow ConvNet (%)	EEGNet (%)
Stressor (%)	60.73	49.14	59.31
Unguided breathing (%)	59.38	56.25	60.38
Guided breathing (%)	53.76	81.07	59.99
Baseline (%)	61.34	64.90	61.18
Average (%)	58.80	62.84	60.21

**Table 3:** Class-wise and overall accuracies for the 1-Layer, 2-Layer, and 3-Layer LSTM RNN classifiers and the hybrid LSTM-FCN classifier

Average accuracy (%)	1-Layer LSTM (%)	2-Layer LSTM (%)	3-Layer LSTM (%)	LSTM-FCN (%)
Stressor (%)	63.95	**90.82**	74.75	57.19
Unguided breathing (%)	62.89	**91.19**	70.43	57.52
Guided breathing (%)	80.09	**94.57**	67.76	72.32
Baseline (%)	73.53	**96.50**	76.07	64.84
Average (%)	70.12	**93.27**	72.26	62.97

Highest classification accuracy are highlighted in bold

### LSTM RNN performance with individual variation

**Table 4:** 2-Layer LSTM RNN classification accuracy and trait anxiety per participant

Participant	2-Layer LSTM RNN classification accuracy (%)	STAI-C trait anxiety score
L2	92.83	37
L3	92.83	32
L4	93.72	24
L5	93.69	30
L6	92.57	33
L7	93.72	32
L8	93.97	46
T1	92.83	25
T2	93.08	37
T4	93.47	42
T5	93.24	28

We were interested in investigating the relationship between the classification accuracy of the two-layer LSTM model (the best performing model) and pre-existing mental conditions. First, we wished to see if there was a significant difference between model accuracy for participants with autism and neurotypical participants. On average, the two-layer LSTM model accuracy for a participant with autism was 93.33%, while the model accuracy for a neurotypical participant was 93.15%. A Mann-Whitney U test was conducted to compare model accuracy for the participants with autism and neurotypical participants and found no significant difference between model accuracy for the two groups (p=0.566), indicating that the two-layer LSTM model performed similarly regardless of whether the participant had autism. We also wished to understand whether an individual’s persistent (trait) anxiety can influence the performance of the two-layer LSTM RNN. We employed Spearman correlation to compare model accuracy and individual STAI-C trait anxiety scores (see Table 4); higher STAI-C scores indicate higher trait anxiety. The analysis yielded a Spearman’s rho of 0.0393, indicating virtually no correlation between trait anxiety and the two-layer LSTM RNN performance.

## Discussion

To the best of our knowledge, in this study we propose for the first time a deep learning-based classifier for decoding mental stress, a complex and covert state, from scalp EEG signals in youth with ASD. Our results show that states of mental stress can be accurately assessed in adolescents with and without ASD as well as in adolescents with varying levels of baseline anxiety. We compared classification accuracy of 4 binary FBCSP-SVM models and 7 multiclass deep learning models. These classifiers were employed to classify the EEG recorded from ASD and neurotypical adolescents performing a task with periods of stress induction (‘Stressor’), resting state (‘Baseline’), guided breathing (‘Guided Breathing’) and unguided breathing (‘Unguided Breathing’). The best classification accuracy was achieved with the multiclass two-layer LSTM at 93.27%.

The 4 binary FBCSP-SVM classifiers performed as follows: 80.45% for ‘Guided Breathing’ vs ‘Stressor’, 87.88% for ‘Baseline’ vs ‘Stressor’, 80.57% for ‘Unguided Breathing’ vs ‘Guided Breathing’, and 78.28% for ‘Unguided Breathing’ vs ‘Stressor’. The FBCSP-SVM performed best when classifying between the pre-task onset resting state epoch (‘Baseline’) and the stress induction (‘Stressor’) conditions, which could be due to the rest periods imposing the least, and the stress condition the most, demands on the participants. Interestingly, the classifier performed relatively well for ‘Unguided Breathing’ vs ‘Guided Breathing’ classes, although these two conditions were similar in terms of stimuli and demands imposed on the participants. Despite our binary FBCSP-SVM classifiers reaching a satisfactory overall classification accuracy of around 82% across all 4 condition pairs, there are several trade-offs pertaining to the use of SVM when compared to deep learning. Although SVMs require less optimizing parameters, these learning models do not suffer from the problem of local minima, and are less computationally demanding than neural networks, they are constrained to a small number of features [87], even when these features are extracted by algorithms [88]. In addition, SVMs cannot consider a robust set of EEG timepoints, rendering them unable to examine the EEG time domain, which is a critical dimension for analyses [88]. Contrastingly, LSTMs are well able to handle temporal information, given their ability to choose to remember or discard information depending on contextual information. Nonetheless, due to their low computational complexity, SVMs remain one of the most popular types of classifiers for EEG-based BCI, in particular for online scenarios. Notwithstanding that adaptive implementations of SVM have been found to be superior to their static counterparts, they often require fully retraining the classifier with new incoming data, resulting in a much higher computational complexity and thus a lack of online applicability [53]. Conversely, with deep learning methods adaptation can be achieved by retraining the input layer with new incoming data. LSTMs in particular are inherently adaptive and thus well suited for real-time scenarios, as their predictions are conditioned by past input. In addition, unlike SVMs, deep learning networks can automatically adjust and optimize their parameters, essentially alleviating the need for feature extraction and requiring far less processing and prior knowledge regarding the original EEG dataset [55, 65]. Lastly, while some multiclass SVMs have been found to outperform neural networks [89], our attempts with multiclass FBCSP-SVMs produced inconsistent results with accuracies ranging between chance-level and 90%.

With regard to the multiclass deep learning models, the Deep ConvNet CNN performed with an overall accuracy of 58.80%, the Shallow ConvNet CNN with 62.84%, the EEGNet CNN with 61.18%, the LSTM-FCN with 62.97%, the one-layer LSTM with 73.53%, the two-layer LSTM with 93.27% and the three-layer LSTM with 72.26%. The high classification accuracies achieved with the LSTM architecture presumably is a result of its ability to learn time dependencies within the data. Indeed, the retention property is useful in mental state monitoring, as considering the past activations of the EEG can drastically improve the prediction of target variables and the brain activity patterns leading up to, and associated with, specific cognitive states. The inherent nature of deep learning models, with hidden layers obscuring intermediate processes occurring within the models, makes it challenging to definitively identify the exact cause for the reduction in performance with the addition of a third LSTM layer. However, it is generally understood that stacking LSTM layers can render the model prone to overfitting [90] as well as the vanishing gradient problem, in which network weights fail to update significantly over time and model training becomes stagnant [91, 92], phenomena that could explain the lower accuracy of the three-layer LSTM. Empirically, it has been shown that a second LSTM layer often provides a significant boost in classification accuracy over a single LSTM [84, 93, 94]; however, the addition of a third LSTM layer or more can have little to no effect on performance [55, 93, 94], and in some cases additional layers can hinder model training and convergence, and in turn degrade performance [91]. Consequently, our leading hypothesis finds that the marginal increase in model complexity between the two-layer and three-layer LSTM further complicated model training while adding comparatively limited improvements, leading to a net loss in model performance.

There are some caveats to consider in the interpretation of our results. First, given that anxiety varies significantly with context and individual, and cannot therefore be induced reliably and equivalently across participants, we utilized mental stress induction via mental arithmetic as a proxy for anxiety. Second, our experiment was designed to induce anxiety as efficiently as possible and thus minimize the time under stress to avoid any undue strain on the participants. Conversely, more time was required for relaxation to set in and the breathing rate to normalize; as a result the time for the mental arithmetic task and the guided or unguided breathing differed. Thus, learning models were trained on an unbalanced dataset, with more ‘Unguided Breathing’ and ‘Guided Breathing’ EEG samples than ‘Stressor’ and ‘Baseline’ samples, with the potential of artificially inflating model accuracy. However, this is unlikely to be a concern for the two-layer LSTM RNN model, which exhibited high sensitivity and specificity metrics across all classes. Lastly, it should be noted that a potential drawback of LSTM RNNs, and of deep learning algorithms in general, is over-reliance upon large datasets. To this regard, the same classifications performed with smaller datasets including only 2 or 3 conditions led to poorer performance (data not shown). However, the experimental 2-layer LSTM accuracy metrics were likely not impacted by a smaller sample size and were indicative of the model’s real-world performance due to every trained model’s very high demonstrated sensitivity, specificity, and predictive ability; we found the 2-layer LSTM models were not only successful in identifying true positives across all classes but also when rejecting false positives in a statistically significant manner in each and every one of our test subjects. Indeed, one major drawback of deep learning is the need for large amounts of data, an issue we aim to remedy in a future study involving a much larger set of participants, both neurotypical and ASD, as well as a more diverse set of stress induction tasks. Given that we have identified a viable classifier for the monitoring of cognitive states related to anxiety, the goal of forthcoming studies will be to refine and validate the two-layer LSTM RNN deep learning model for prospective implementation in a personalized pBCI. Such a system will be capable of monitoring for periods of stress and hone in on an individual’s optimal respiration patterns by adapting the breathing entrainment parameters in a closed-loop manner.

In summary, the goal of this study was to compare several learning models or classifiers on their ability to assess mental stress levels from EEG recordings performed on adolescent students to determine the feasibility of an EEG-based BCI capable of real-time identification and the mitigation of anxiety through optimized respiration entrainment. Of the different classifiers we compared, two-layer LSTM yielded the highest classification accuracy (93.27%), opening new avenues of decoding covert mental states for BCI-based neuroadaptive applications to benefit youth with autism.

## Data Availability

The informed consent forms signed by the study participants and their legal guardians assured anonymity and utmost confidentiality of participant data, so it is not possible to make the data publicly available at this time.
